# A new method for determining physician decision thresholds using empiric, uncertain recommendations

**DOI:** 10.1186/1472-6947-10-20

**Published:** 2010-04-08

**Authors:** Michael V Boland, Harold P Lehmann

**Affiliations:** 1 Wilmer Eye Institute, Johns Hopkins University, Baltimore, MD, USA; 2 Division of Health Sciences Informatics, Johns Hopkins University, Baltimore, MD, USA

## Abstract

**Background:**

The concept of risk thresholds has been studied in medical decision making for over 30 years. During that time, physicians have been shown to be poor at estimating the probabilities required to use this method. To better assess physician risk thresholds and to more closely model medical decision making, we set out to design and test a method that derives thresholds from actual physician treatment recommendations. Such an approach would avoid the need to ask physicians for estimates of patient risk when trying to determine individual thresholds for treatment. Assessments of physician decision making are increasingly relevant as new data are generated from clinical research. For example, recommendations made in the setting of ocular hypertension are of interest as a large clinical trial has identified new risk factors that should be considered by physicians. Precisely how physicians use this new information when making treatment recommendations has not yet been determined.

**Results:**

We derived a new method for estimating treatment thresholds using ordinal logistic regression and tested it by asking ophthalmologists to review cases of ocular hypertension before expressing how likely they would be to recommend treatment. Fifty-eight physicians were recruited from the American Glaucoma Society. Demographic information was collected from the participating physicians and the treatment threshold for each physician was estimated. The method was validated by showing that while treatment thresholds varied over a wide range, the most common values were consistent with the 10-15% 5-year risk of glaucoma suggested by expert opinion and decision analysis.

**Conclusions:**

This method has advantages over prior means of assessing treatment thresholds. It does not require physicians to explicitly estimate patient risk and it allows for uncertainty in the recommendations. These advantages will make it possible to use this method when assessing interventions intended to alter clinical decision making.

## Background

The use of thresholds has been considered as a means of making medical decisions for over 3 decades [1-3]. To utilize this method, physicians must be able to estimate the baseline risk of each individual patient. Knowing the risk of the patient, the physician must also be able to determine thresholds for risk above which they will order additional testing or initiate treatment. Furthermore, the physician must be able to determine how a patient's risk will change based on the result of a particular test. If the test has no chance of moving the patient's risk above the treatment threshold, for example, then the test should probably not be ordered.

There is, however, a body of evidence that documents physicians' inaccuracy in predicting the risk of individual patients [4-8] and specifically in patients with ocular hypertension[9]. There is also evidence that clinicians do not use the threshold method when making decisions [10] and that intuitive threshold estimates do not match observed thresholds for treatment[11]. Given these problems with the estimation of *a priori *risk levels, the threshold method becomes suspect as a prescriptive means of rational decision making. On the other hand, the concept of risk thresholds is potentially very useful for comparing physicians to one another or for comparing physicians to proposed rational standards. It may therefore be useful to consider these descriptive uses of thresholds differently from the problematic prescriptive methods mentioned above.

One approach to determining physician treatment thresholds is to provide the clinicians with case scenarios and then ask them for an estimate of the probability of disease and for their recommendation to treat or treat. By varying the risk of disease represented by the case scenarios, it is possible to identify a clinician's risk thresholds for either ordering additional testing or initiating treatment[12]. This method still requires clinicians to estimate probabilities and because of this requirement, it becomes very difficult to compare the threshold derived from one physician to that of another. Furthermore, there is no guarantee that the risk estimates are based on any rational synthesis of evidence. Finally, throughout the history of eliciting risk estimates, physicians have both been uncomfortable in providing numerical values and have expressed the need to communicate the uncertainty in those estimates[13].

Another approach to threshold determination removes the need for clinicians to explicitly estimate any risk whatsoever. Plasencia *et al. *described a method in which they used binary logistic regression to estimate treatment thresholds[14]. Their method depends on another advance in risk estimation, the synthesis of large population based studies to produce risk calculators that can be used to estimate disease risk for a particular patient. The Framingham study provides a good example of how risk factors found in epidemiologic studies can be combined to summarize the risk of an individual [15-17]. Since the risk associated with each patient can now often be estimated based on published evidence, it is no longer necessary to ask the clinicians for their estimate of that risk. Instead, they can be asked for their treatment recommendation only. Building on the work of Hartz *et al.*, [18] Plasencia and others used binary logistic regression to estimate treatment thresholds[14]. This method has the advantage of using the same risk for each case scenario rather than using the many intuitive estimates provided by clinicians. In this way, it is possible to compare decision thresholds across physicians.

One disadvantage of the logistic regression method is that it requires concrete binary decisions be made. Because uncertainty is an important component of clinical decision making,[19] we developed a method for assessing treatment thresholds that allowed clinicians to make explicit use of their uncertainty. Given a situation in which we can estimate the risk of glaucoma for any patient with ocular hypertension, we set out to determine whether ordinal (as opposed to binary) logistic regression could be used to derive physician treatment thresholds from treatment recommendations made on a set of clinical scenarios. By allowing for more than two levels of recommendation (yes or no), we are also allowing for physicians to express uncertainty.

As opposed to providing quantitative estimates of patient risk, clinicians routinely make recommendations that are presumably driven by that same patient risk, but implicitly so. We developed the proposed method to take advantage of this fact and to derive some estimate of physician interpretation of risk, as reflected by their explicit treatment recommendations. There is clearly still variability in physician behavior but the approach allows us to analyze usual physician behavior (treatment recommendations) rather than unusual behavior (assigning quantitative risk).

While the literature is clear about clinicians' inability to quantify risk, the literature has little to say about the ability of clinicians to state accurately whether a given patient is above or below that risk. In fact, there is reason to believe, given their general successful functioning, that they are good at this task. It is this task that we have quantified. As a first attempt at assessing this skill, we have created a de facto random effects model, where each clinician has their own threshold (and ability to compare with respect to that threshold) sampled, in turn, from a population threshold; it is that latter threshold that we estimate with our regression model. Furthermore, because prior research is clear that physicians are unable to reliably assign a numeric value to a particular patient's risk of a particular disease or outcome, we employed a method to *discover *the implicit risk levels at which physicians change their behaviour. This method will then let us compare the impact of interventions like risk calculators, clinical decision support tools, and physician education.

To evaluate our method, we chose to use ocular hypertension (elevated eye pressure) as the condition under study. Ocular hypertension is a known risk factor for developing glaucoma, a significant cause of blindness worldwide[20]. Because of its association with glaucoma, ocular hypertension was the subject of a large clinical trial, the Ocular Hypertension Treatment Study (OHTS)[21]. The OHTS randomized over 1600 patients with elevated eye pressure to either pressure lowering treatment or observation and then monitored both groups for development of glaucoma. Prior to the OHTS, it was not clear which patients with ocular hypertension would benefit from treatment to prevent glaucoma. Analysis of the study has since clarified the risk factors associated with the conversion from ocular hypertension to glaucoma. Among the most important outcomes of the OHTS have been risk calculators that can be used to estimate the risk of developing glaucoma in individual patients [22-24]. Such calculators may be able to help physicians identify those patients most at risk of developing glaucoma and are important as an objective measure of risk in the method we describe. Although we chose to evaluate this method using ocular hypertension as the disease in question, this approach will be applicable to any disease process for which experimenters can calculate the risk of an outcome for each patient presented to the physicians under study. Examples include cardiovascular risk or cancer mortality.

## Results

In brief, the method we propose to analyze treatment recommendations allows physicians to express their recommendations using an ordinal scale that includes uncertainty. The seven-point scale used here ranged from 'Definitely No' (no treatment) to 'Definitely Yes'. These responses are then analyzed using ordinal regression to estimate the point of maximal uncertainty for each physician.

This work was approved by the institutional review board of the Johns Hopkins University School of Medicine and adhered to the tenets of the Declaration of Helsinki.

### Algorithm

**Figure 1:**
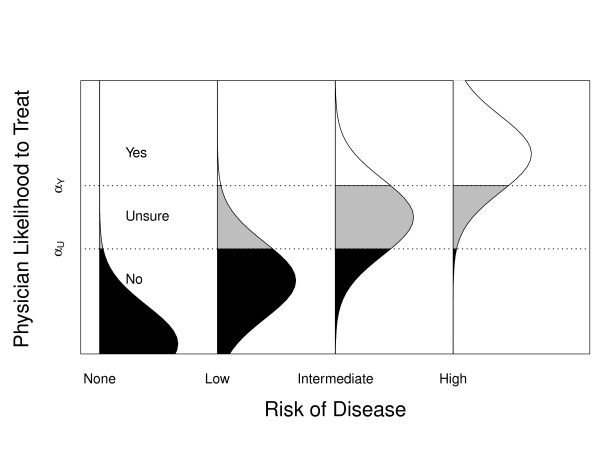
**A graphical representation of the role of ordinal regression in this method**. The four risk levels are arbitrary and chosen for illustrative purposes only. The normal distribution shown at each risk level represents the assumed underlying continuous probability of treatment recommendation that is being assessed using the ordinal scale (Yes, Unsure, No). The two thresholds (α_U_, α_Y_) are the intercepts derived from ordinal regression analysis of physician responses and form the boundaries between the three response levels.

The role of ordinal regression in this method is depicted graphically in Figure 1. Four arbitrary levels of disease risk are shown. For each level of risk, it is assumed that there is a continuous variable representing the likelihood of physicians recommending treatment. The vertically oriented normal distributions depict the distribution of this underlying variable at each level of risk. As disease risk increases, this distribution shifts upward, indicating that physicians are more likely to recommend treatment. Since this continuous variable cannot be measured directly, however, it is modelled in practice by an ordinal ranking (Yes, Unsure, No). For the purposes of this method, this ordinal variable represents physician likelihood to recommend treatment. The two intercepts in the ordinal regression model (α_U_, α_Y_) represent the break points between the three levels of the ordinal scale. The point we have defined as the treatment threshold is the level of risk at which the likelihood of a Yes recommendation and a No recommendation are equal. This point is represented at the "Intermediate" risk level in the figure.

From the definition of ordinal regression,[25,26] the following relationship exists between the treatment recommendation (*t*) and the patient's risk of disease (*r*), where j is the level of recommendation on the ordinal scale (No, Unsure, Yes), α_j _is the intercept coefficient for that level, β is the regression coefficient for risk, and *P() *indicates probability.(1)

To estimate each physician's threshold for recommending treatment, we are interested in the value for risk at which they are equally likely to make a Yes or No recommendation (i.e., are maximally unsure).(2)

Using the fact that the possible recommendations, No, Unsure, and Yes are ordinal, we know that the probability of a No recommendation is the probability of a recommendation greater than or equal to No minus the probability of a recommendation greater than or equal to Unsure. Since there are no recommendations less than No, the probability of a recommendation greater than or equal to No is 1.(3)

Furthermore, we know that the probability of a Yes recommendation is equal to the probability of a recommendation greater than or equal to Yes since there are no recommendations beyond Yes.(4)

Combining Equations 1,3, and 4, we can generate expressions for the probability of Yes and No recommendations in terms of risk and the ordinal regression coefficients.

Setting these two equations equal to each other and solving for risk produces the following:

For each participating physician we performed ordinal regression on their treatment recommendations and then calculated *r *as an estimate of their treatment threshold. For physicians who only used No and Yes in their recommendations (2 physicians), we used logistic regression in the same way described by Plasencia. The instances where physicians use only two categories of response are really special cases of the full three level problem in that those physicians are never unsure of their decisions. By defining the threshold for recommending treatment to be the point at which the likelihoods of Yes and No recommendations are equal, we are really using the same model for both groups while still allowing the majority of physicians to express uncertainty. Finally, for those clinicians who never recommended treatment (3 physicians), we assigned a threshold equal to the average of the highest calculated risk among the simulated cases and 100%. This estimates the risk threshold for this subgroup of physicians as falling between the highest risk in our cases (which was too low for them to recommend treatment) and the highest possible risk (100%). Although this choice is somewhat arbitrary, we believe this approach is appropriate given that we know these physicians would probably not treat patients with even the highest risk in our case scenarios and also know that they sometimes recommend treatment of ocular hypertension (they denied that they "never" treated these patients as part of the study). Threshold values were calculated for each physician rather than for the entire group in order to capture the expected differences in their likelihood to treat ocular hypertension. This within-subject analysis is supported by prior work using expectancy-value models[27].

### Testing

To simulate the patient population of the OHTS, 50 cases of ocular hypertension were generated using the summary statistics of the OHTS population [28]. Each simulated case was a list of patient attributes instantiated with specific values. The attributes known from the OHTS study to be risk factors for initial glaucoma are age, intraocular pressure (IOP), cup-disc ratio (CDR), pattern standard deviation (PSD) from automated field testing, central corneal thickness (CCT), and self-reported diabetes mellitus. We chose to use only the known risk factors for the development of glaucoma so that, given a sample size of 50 physicians, we could avoid under-determined multivariate models. We created scenarios by generating values from the published proportions, means and standard deviations from the OHTS population. Values for the continuous parameters were drawn from normal distributions. Where appropriate, values of risk factors in our scenarios were restricted based on OHTS inclusion and exclusion criteria [29]. The estimated 5-year risk of glaucoma was then calculated for each scenario using the equation described by Medeiros *et al. *[23] The model underlying this calculator produced a concordance index between 0.68 and 0.73, though they did not report any other statistics indicating how much of the true variability in risk their model was able to explain.

To recruit participants, we sent two email messages to members of the American Glaucoma Society (AGS) over the course of two weeks. Although the AGS was not able to confirm the number of active participants on the society e-mail list, the total membership was approximately 500 at the time of the survey. To prevent bias in recruitment of physicians, recipients were not told the specific nature of the study, only that they would be reviewing cases of ocular hypertension. Because of the estimated time required to complete the study (30-45 minutes) and to further reduce any selection bias, they were also offered compensation of $150 for their time. Enrolment in the study was stopped when we surpassed the target of 50 subjects.

Once they accessed the study web site, physicians were provided with a brief consent document. If they agreed to participate, they were first asked questions intended to identify and exclude physicians who either *always *or *never *treat ocular hypertension. Physicians who did not report using intraocular pressure as the sole criteria for treatment were then asked to review 50 case scenarios without being given the estimated risk of conversion to glaucoma. Participants were instructed to review the scenarios that included values for all six OHTS-derived risk factors plus patient gender. For each simulated patient, the physicians were asked how likely they would be to recommend treatment to prevent glaucoma. Participants were explicitly instructed that this recommendation represented their initial position in their discussion with the patient. Responses were recorded along a seven-point scale that included 'Definitely No' (no treatment), 'Probably No', 'Possibly No', 'Unsure', 'Possibly Yes', 'Probably Yes', and 'Definitely Yes' with the goal of collapsing them into three groups for the purpose of analysis.

After completing the study, participants were all asked to provide their gender, racial background, number of years in practice, subspecialty training, monthly clinic volume, fraction of practice devoted to glaucoma, and use of a risk calculator in their practice.

As the first step in analyzing the physician responses, the seven-point treatment recommendation scale was collapsed into three levels. 'Definitely No' and 'Probably No' were combined as 'No'. 'Possibly No', 'Unsure', and 'Possibly Yes' were combined as 'Unsure', while 'Probably Yes' and 'Definitely Yes' were labelled 'Yes'. The choice of a 3-point scale was made to facilitate interpretation of the subsequent regression analysis and to ensure an adequate number of responses in each category. The particular mapping was chosen to move responses from the 7-point scale to the nearest value in the 3-point scale while still explicitly maintaining the concept of "unsure". There is evidence that 3-point scales are adequate [30] and this approach is suggested for Likert-scale data in order to make subsequent analysis more understandable[31,32]. The treatment recommendations were then analyzed using ordinal regression as described above to identify the treatment threshold for each physician.

### Implementation

**Figure 2:**
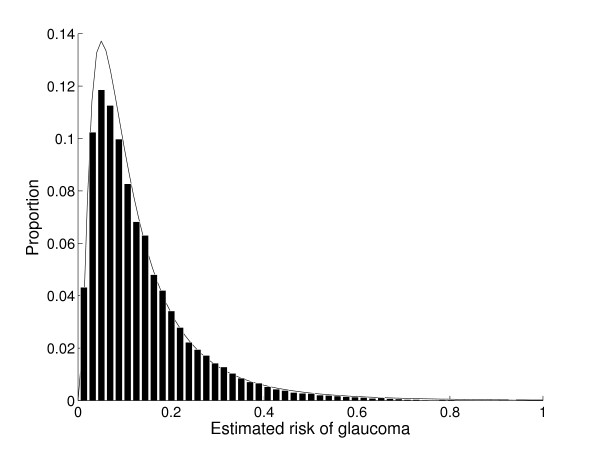
**The distribution of the 5-year risk of glaucoma**. The distribution of the 5-year risk of glaucoma in the population from which the 50 simulated cases were drawn. The solid line is a log-normal distribution with the same mean and variance of risk as the simulated cases.

The distribution of glaucoma risk in our simulated population is shown in Figure 2. Risk is distributed in a truncated log-normal fashion and this simulated population is similar to a population of patients reported by Medeiros *et al. *as part of the Diagnostic Innovations in Glaucoma Study (DIGS) [23]. The DIGS population had an average predicted risk of 14.3% while our simulated cases had an average predicted risk of 13.8%. Furthermore, the actual glaucoma risk of those DIGS subjects with a predicted risk above 15% was 26%, which is identical to the risk of the same group in our simulated population. The simulated cases therefore represent a good approximation to "real" patients as they were created using statistics for risk factors derived from the results of a clinical trial (OHTS) and because the overall distribution of glaucoma risk is the same as that in a cohort study (DIGS).

The physicians participating in the study were all members of the AGS and their demographic attributes are summarized in Table 1. The fact that these subjects were drawn from a subspecialty group is reflected both in the high percentage of subspecialty training and in the percentage of their practice devoted to glaucoma. This group is therefore not representative of most ophthalmologists. Because membership demographics are not available from the AGS, it is not possible to determine the degree to which these participants were representative of the organization as a whole. Of the 58 participants in the study, two of them indicated that they treat all patients with eye pressure above 21 mmHg. Because these two physicians were not using the overall risk of glaucoma as the basis for their treatment recommendations, they were excluded from further analysis.

**Table 1:** Demographics of study participants

	Number [%]
Gender (Male)	48 [83]
Racial Background	
Asian	9 [16]
Black	2 [3.4]
White	41 [71]
Other or None	6 [10]
Glaucoma Training	57 [98]
Risk Calculator Use	
Never	31 [53]
Sometimes	25 [43]
Always	2 [3.4]
	Mean (std. dev.)
Length of practice (years)	16.8 (10.2)
Patients seen per month	444 (217)
Percentage of Practice Devoted to Glaucoma	77 (20)

All values are derived from self-reported data provided by study participants (N = 58). Glaucoma Training refers to the number of participants who had subspecialty training and Risk Calculator Use indicates how frequently the participants used one of the available risk calculators in their practice.

**Figure 3:**
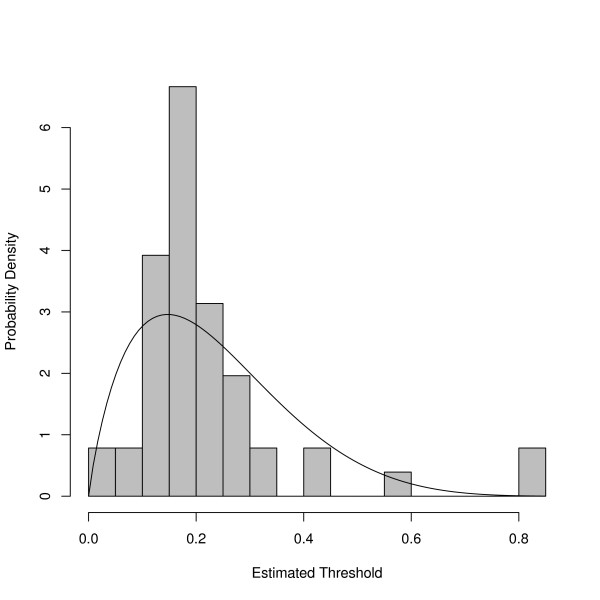
**The distribution of estimated treatment thresholds for glaucoma specialists**. The solid line shows a beta distribution with coefficients α = 2.56 and β = 9.14. The coefficients of the beta distribution were obtained using a maximum-likelihood method (fitdistr in R) and have standard errors of 0.48 and 1.83 respectively.

Using the threshold estimation method described above, a treatment threshold was calculated for each of the 56 remaining physicians using ordinal regression as described in Methods (Figure 3). The average treatment threshold over all 56 physicians was a 23% chance of conversion to glaucoma over five years. Because the distribution of derived treatment thresholds was skewed to the right, however, the most common values fell between 10 and 20% which are similar to suggested treatment thresholds derived from expert opinion (15% 5 year risk)[33] and from decision analysis (10% 5 year risk)[34]. The distribution of thresholds also captures the fact that some physicians would treat all or almost all of the cases to prevent glaucoma (far left in Figure 3) and that some would treat none or almost none of them (far right in Figure 3).

Statistics were calculated for the ordinal regression model calculated for each physician. The average R^2 ^value (using the method of Nagelkerke) was 0.40 with a standard deviation of 0.14. The concordance index, which is the same as the area under the curve in ROC analysis, had an average value of 0.81 with a standard deviation of 0.06. This value for concordance is actually better than that for the risk calculator model mentioned above (0.68 to 0.71).

## Discussion

We have defined and applied a new method for assessing treatment thresholds that accommodates uncertainty in physician treatment recommendations. In settings for which patient risk can be objectively estimated, this method provides estimates of the risk threshold for individual physicians using only their treatment recommendations. Like Plasencia *et al.*, our method avoids the problem of asking physicians for explicit estimates of patient risk. This feature is important, as there is now a body of work supporting the contention that physicians are, as a group, not effective at making such estimates. In contrast to Plasencia *et al.*, our method is not restricted to binary (yes/no) treatment recommendations. By allowing for uncertainty on the part of physicians, the method described above is more reflective of medical decision making[19].

Using simulated cases of ocular hypertension to evaluate our method, we found that the participating physicians were most likely to recommend treatment at a risk level supported by expert opinion or by formal decision analysis. There was, however, a wide range of risk thresholds with some physicians recommending treatment in most cases and some recommending treatment in almost none. Such inter-physician variability is consistent with that of Eisenberg and Hershey who derived treatment thresholds using physician risk estimates[12].

We also found that the combination of physician responses and our model showed good prediction of the estimated risk, at least as measured by the concordance index. The R^2 ^values calculated for each physician's ordinal regression model did suggest that there is a significant proportion of risk that is not explained by physician recommendations, however. This problem is not inherent in our model and is more likely due to variability in physician ability to estimate risk, either explicitly or through their clinical decisions.

One trade-off made in the design of our study was the use of simulated cases rather than "real" patients. While it is possible to argue that this reduces the ability to generalize our results, we made every effort to ensure our cases were as representative as possible. Specifically, we used the demographic and risk factor statistics from the OHTS to generate our cases and we compared the risk profile from our population to that of a cohort study independent of the OHTS. For these reasons, and because we had to present only numeric data to the participating physicians, we believe our cases were representative of cases of ocular hypertension available in clinic.

Alternative approaches to analyzing response data include both item response theory and Rasch analysis. Both of these methods are intended for analysis of events (case scenarios or test questions) across individuals. Because the quantity we are interested in here is some measure of risk tolerance by physicians, we needed a method that described responses within a respondent across those events (case scenarios). For this reason, we did not feel item response or Rasch theory was most appropriate.

This approach to estimating physician treatment thresholds has a potential role in the evaluation of interventions designed to modify physician behaviour. For example, one might be interested in assessing the impact of a decision support tool on the treatment recommendations made by physicians. Such tools include the risk calculators that are now available for ocular hypertension and other conditions, providing the clinician with an explicit estimate of patient risk. By calculating risk thresholds for recommendations made both with and without the support tool, it will be possible to determine how the tool impacts physician decisions in terms of how much risk of disease they are willing to tolerate before recommending treatment. As a numeric value, these risk thresholds are also amenable to a variety of analyses that might not be possible with more subjective measures. Elicitation of decision thresholds in this way may also lead to better customization of decision support systems, which may, in turn, improve adoption[35].

In future studies, the threshold we calculate will be used as an outcome measure. For instance, to assess the impact of a decision support tool on clinicians, we will be able to assess the (implicit) threshold before and during/after use of such a calculator. Similarly, we will assess the impact of patient characteristics on the implicit threshold, and to see if characteristics beyond those that are evidence-based have an effect on clinical decisions. Such discoveries may help explain treatment variability among ophthalmologists or other clinicians.

## Conclusions

In summary, we have described a new method for assessing physician risk thresholds using only empiric, uncertain treatment recommendations. This method has advantages over prior work in that it does not require physicians to estimate risk directly and allows them to be uncertain in their recommendations. We have tested this method using simulated cases of ocular hypertension and found wide variability in physician risk thresholds with the most common values near thresholds suggested by expert opinion and decision analysis. Based on the characteristics of the method, we believe it has potential as a means of assessing the impact on physician recommendations of decision support tools.

## Competing interests

The authors declare that they have no competing interests.

## Authors' contributions

Both authors contributed to the design of the methods described above, to the analysis of the evaluation study, and to the writing of the manuscript. MB designed and carried out the evaluation study. Both authors read and approved the final version of the manuscript.

## Pre-publication history

The pre-publication history for this paper can be accessed here:

http://www.biomedcentral.com/1472-6947/10/20/prepub
